# Multi-Omics Analysis Identified *LTB4R* as a Peripheral Blood Diagnostic Biomarker for Colorectal Cancer

**DOI:** 10.3390/ijms27062575

**Published:** 2026-03-11

**Authors:** Tong Wang, Changqing Li, Zongkui Wang

**Affiliations:** Institute of Blood Transfusion, Chinese Academy of Medical Sciences & Peking Union Medical College, Chengdu 610052, China; 15883000114@163.com

**Keywords:** colorectal cancer, scRNA-sequencing, bioinformatic, *LTB4R*, biomarker

## Abstract

Colorectal cancer (CRC) is a prevalent malignant tumour, with its incidence and mortality rates consistently ranking among the highest and exhibiting an upward trend. Extensive screening and early diagnosis are crucial for managing CRC progression and improving patient prognosis. This study aims to construct a novel analytical framework for integrating the sequencing data from tumour tissue and peripheral blood. By integrating and analysing the multi-omics data and clinical data from tumour tissues and peripheral blood, we confirmed that the *LTB4R* gene is significantly upregulated not only in tumour tissues but also in the peripheral blood of CRC patients. Further single-cell RNA sequencing (scRNA-seq) and immune cell correlation analyses revealed that *Leukotriene B4 receptor 1* (*LTB4R*) is primarily expressed in macrophages, T cells, and other immune cells, with a significant negative correlation observed with M1-type macrophages, suggesting its potential pro-tumourigenic role in CRC by suppressing M1 macrophage. Additionally, simulated gene knockout analysis (scTenifoldKnk) demonstrated that *LTB4R* knockout significantly impacts immune-related pathways, including immune response and immune receptor activity. These findings not only highlight the potential of *LTB4R* as a peripheral blood diagnostic marker for CRC but also elucidate its involvement in tumour progression, offering novel insights for early clinical diagnosis and tumour screening systems.

## 1. Introduction

Colorectal cancer (CRC) ranks among the malignancies with the highest incidence and mortality rates globally, positioning third in incidence and second in overall mortality [1]. Epidemiological data indicate that CRC accounted for 1.9 million new cases in 2020, with projections surpassing 3 million by 2040 [2]. Although existing screening strategies have moderately reduced mortality rates, most patients are diagnosed at advanced stages, underscoring the urgent need to develop diagnostic biomarkers and optimise screening modalities [3]. Currently, CRC clinical diagnosis primarily relies on colonoscopy and histopathological examination, supplemented by imaging assessments and a limited panel of serum biomarkers (e.g., CEA, CA19-9) [4,5]. However, these methods exhibit limitations in early lesion detection, micrometastasis identification, and prognosis prediction, while also lacking specific biomarkers to guide personalised treatment decisions [6,7,8]. Given the escalating disease burden and unmet clinical needs, identifying novel biomarkers holds significant potential to enhance diagnostic accuracy, improve patient outcomes, and facilitate community-based screening.

In recent years, the expansion of public databases and breakthroughs in bioinformatics have opened new avenues for systematically screening key molecular entities in disease pathogenesis [9]. scRNA-seq has revolutionised research into the tumour microenvironment (TME), enabling the high-resolution analysis of cellular heterogeneity [10,11]. Furthermore, scRNA-seq facilitates the identification of distinct macrophage subpopulations and elucidates their functional roles across various solid tumours [12]. By integrating multi-omics datasets or designing new data analysis frameworks (including single-cell RNA sequencing, peripheral blood RNA sequencing, and batch RNA sequencing), we can efficiently extract disease-specific molecular features from the vast amount of biological information, thereby deepening our understanding of the causes and progression of diseases and identifying more biologically relevant biomarkers with greater potential for clinical applications, which might be used for early clinical diagnosis.

In this study, we initially identified differentially expressed genes (DEGs) through a comparative analysis of CRC tissues versus normal tissues. Subsequently, survival analysis and prognostic assessment of these DEGs were performed to screen for candidate genes, with *LTB4R* emerging as a priority. We then conducted further analysis of single-cell sequencing data to construct intercellular communication networks among immune cells, alongside evaluating the distribution of *LTB4R* within immune cell populations to delineate its expression patterns. To elucidate the functional impact of *LTB4R*, we employed in silico gene knockout modelling to pinpoint the primary signalling pathways and key molecular entities affected by its depletion. Additionally, single-factor survival analysis, multi-factor survival analysis, receiver operating characteristic (ROC) curve assessment, and immune cell correlation analysis were utilised to validate *LTB4R*’s diagnostic potential and its capacity for the negative regulation of M1-type macrophages. Finally, we analysed peripheral blood RNA sequencing samples from CRC patients and healthy controls, revealing *LTB4R*’s potential as a liquid biopsy biomarker and underscoring its significant clinical utility.

By integrating cutting-edge bioinformatics approaches with multi-omics datasets, this study not only delineates the pivotal role of *LTB4R* in CRC pathogenesis but also unveils, for the first time, its substantial potential as a diagnostic biomarker for CRC. These findings provide valuable insights for researchers and clinicians in the field of precision medicine.

## 2. Results

### 2.1. Subsection Identified Key Genes Related to CRC

**Figure 1:**
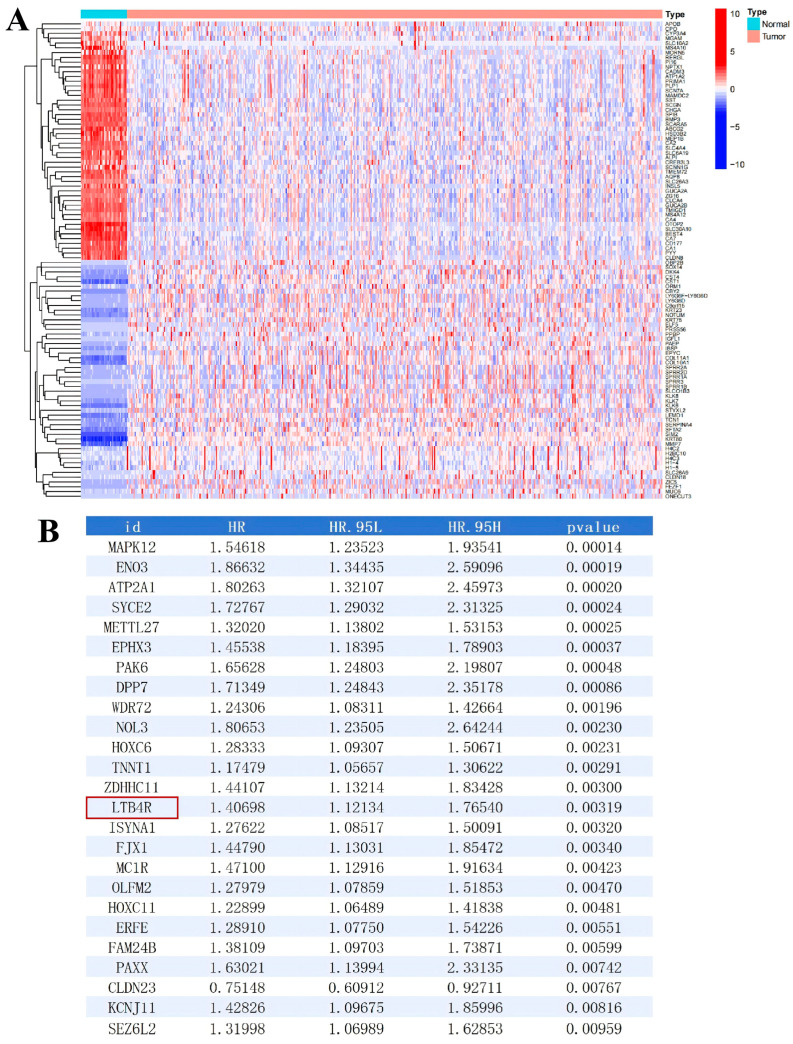
Screening of differentially expressed genes in colorectal cancer. (**A**) Heatmap of differentially expressed genes derived from the COAD data downloaded from the TCGA database (tumour group versus normal group). (**B**) List of differentially expressed genes obtained after survival analysis and independent prognostic analysis.

We initially analysed RNA-sequencing data and clinical data of CRC from the TCGA database to identify significant gene expression differences between CRC and healthy tissues. As illustrated in Figure 1A and listed in Table S1, distinct gene expression profiles were observed between CRC and control groups. A total of 3724 DEGs (2632 genes were upregulated, while 1092 genes were downregulated) were identified, with log2 fold change (log2FC) > 0.585 and false discovery rate (FDR) < 0.05. Next, we conducted a survival analysis on the differentially expressed genes based on the clinical data and found that 109 genes showed significant differences (as listed in Table S2). Subsequently, we conducted an independent prognostic analysis on the DEGs identified through the survival analysis. The results showed that 58 genes (including *MAPK12*, *ENO3*, *ATP2A1* and *PAK6*, etc.) could serve as independent prognostic factors for CRC (as listed in Table S3; the top 25 genes are shown in Figure 1B). These top 25 genes were selected as candidate genes for subsequent research.

### 2.2. LTB4R Gene Was Highly Expressed in the Peripheral Blood of Patients with CRC

**Figure 2:**
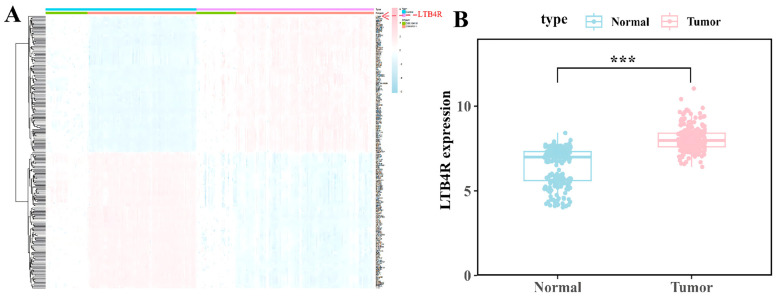
The *LTB4R* gene is highly expressed in the peripheral blood of colorectal cancer patients. (**A**) Heatmap of differentially expressed genes in the GEO dataset of peripheral blood samples. (**B**) Violin plot of the expression levels of the *LTB4R* gene in the peripheral blood of colorectal cancer patients and healthy individuals. ***(*p* < 0.001).

The analysis results of the above TCGA database were derived from intestinal tumour tissue samples. Due to the difficulty in obtaining intestinal tissue samples in actual clinical analysis, this limits its ability for early diagnosis and large-scale screening. However, peripheral blood samples, due to their accessible acquisition method, were the preferred choice for developing biomarkers with high clinical application potential. Therefore, we further analysed the differentially expressed genes in peripheral blood samples (Figure 2A). Subsequently, we selected the genes that were differentially expressed in both tumour tissues and peripheral blood as potential candidate genes. Moreover, by consulting the literature and removing genes that have been widely reported as CRC markers (such as *ENO3*, *MAPK1*, *SYCE2*, etc.), we confirmed the *LTB4R* gene (as shown in Figure 2B, the *LTB4R* gene was highly expressed in the peripheral blood of CRC patients) as the key candidate gene for this study and used it for subsequent analysis and research. These results demonstrated the potential of *LTB4R* as a diagnostic marker for CRC.

### 2.3. Single-Cell Sequencing Analysis Revealed the Distribution of LTB4R in Immune Cells

**Figure 3:**
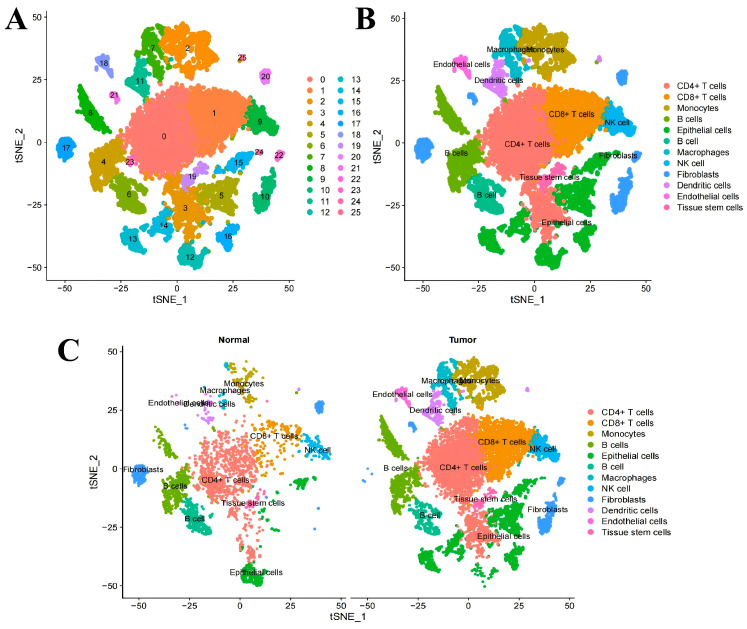
Graphical representation of single-cell sequencing data analysis results. (**A**) Clustering analysis diagram, (**B**) Cell annotation diagram, (**C**) Principal component analysis diagram obtained from single-cell analysis, as well as the comparison diagram between the tumour group and the control group.

We downloaded the CRC single-cell sequencing data from the GEO database (GSE231559) for analysis. First, a series of routine analyses were conducted, including quality control analysis of single-cell sequencing data, sequencing depth analysis, differential gene analysis, principal component analysis, etc. (Figure S1). The results of cluster analysis showed that all the cells were clustered into 25 cell groups (Figure 3A), including CD4+ T cells, CD8+ T cells, macrophages, monocytes, NK cells, epithelial cells, etc. (Figure 3B). And the principal component analysis graphs of the tumour group and the normal group respectively show similar cell clustering (Figure 3C).

**Figure 4:**
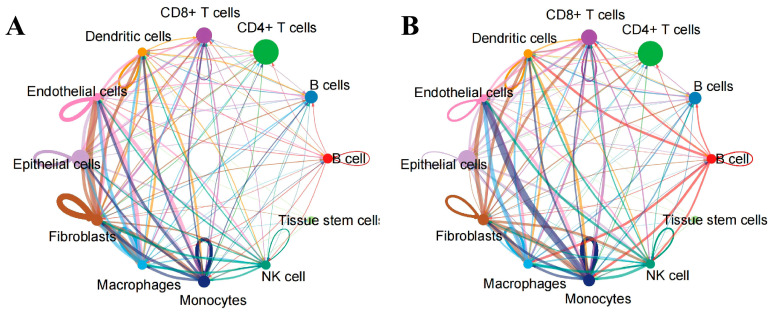
Interaction map of single-cell analysis. The size of the nodes in the figure indicates the number of cells. The colour of the lines in the figure represents the colour of the ligand cells. (**A**) Interaction number map. The thicker the line, the more cells involved in the interaction. (**B**) Interaction strength map. The thicker the line, the higher the strength of the interaction.

**Figure 5:**
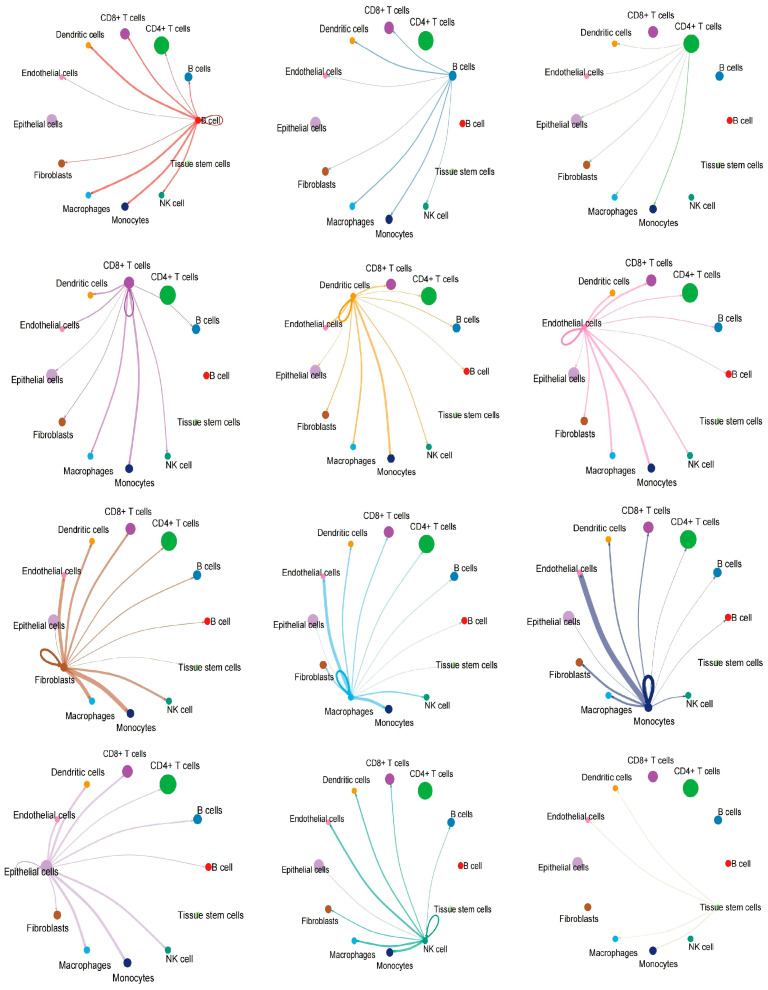
Cell interaction map from single-cell analysis. Each small figure represents the interaction between different cell populations as ligands and other cell populations (as receptors).

Subsequently, we conducted a cell communication analysis to investigate the signal transduction mechanisms between different types of cells. Each node in the figure represents a type of cell, and the larger the node, the greater the number of cells. The results show that the interactions between Endothelial cells, Epithelial cells, Fibroblasts cells and other cells were the most numerous (Figure 4A). The Monocytes cells, Fibroblasts cells, Macrophages cells and other cells have the strongest interaction intensity with each other (Figure 4B). Furthermore, the detailed graphical results also provide a clearer illustration of the intercellular interaction pattern where this cell acts as the ligand and the connected cells serve as the receptors (Figure 5).

**Figure 6:**
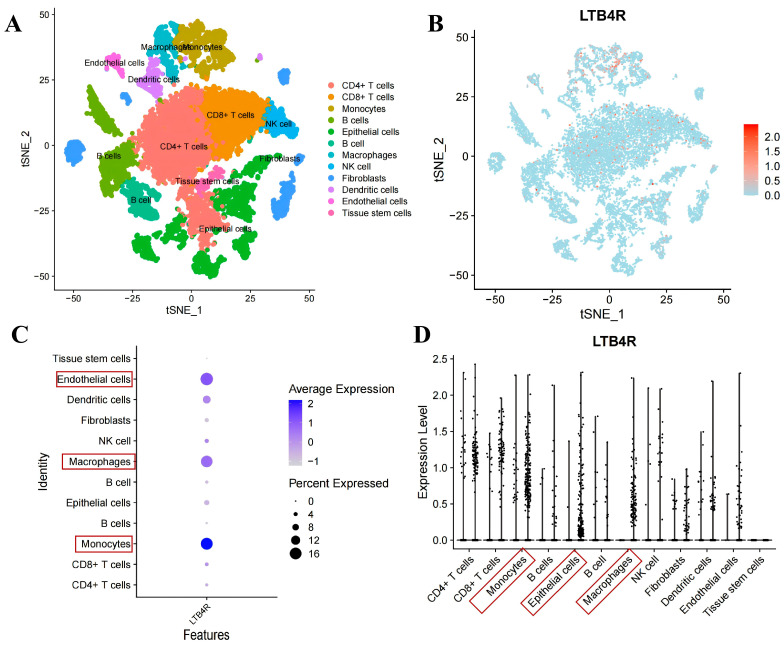
Expression of the *LTB4R* gene in different immune cells. (**A**) Clustering diagram of single-cell analysis. (**B**) Expression distribution map of *LTB4R* in different cell subpopulations. (**C**) Correlation analysis diagram of *LTB4R* with different immune cells. (**D**) Expression pattern of *LTB4R* in different immune cells.

To clarify the relationship between *LTB4R* expression and cell subpopulations, we analysed the distribution of this gene in different cell subgroups. The results of principal component analysis showed that the *LTB4R* gene was mainly expressed in monocytes, macrophages, epithelial cells, CD4+ T cells and CD8+ T cells, etc. (Figure 6A,B). Furthermore, the results of the correlation analysis and the expression analysis also indicate that the *LTB4R* gene is mainly expressed on monocytes, macrophages and epithelial cells (Figure 6C,D). These results indicate that, in the CRC tissue, *LTB4R* was closely associated with monocytes, macrophages and epithelial cells.

### 2.4. Simulated LTB4R Knockout Revealed Its Underlying Molecular Mechanism

**Figure 7:**
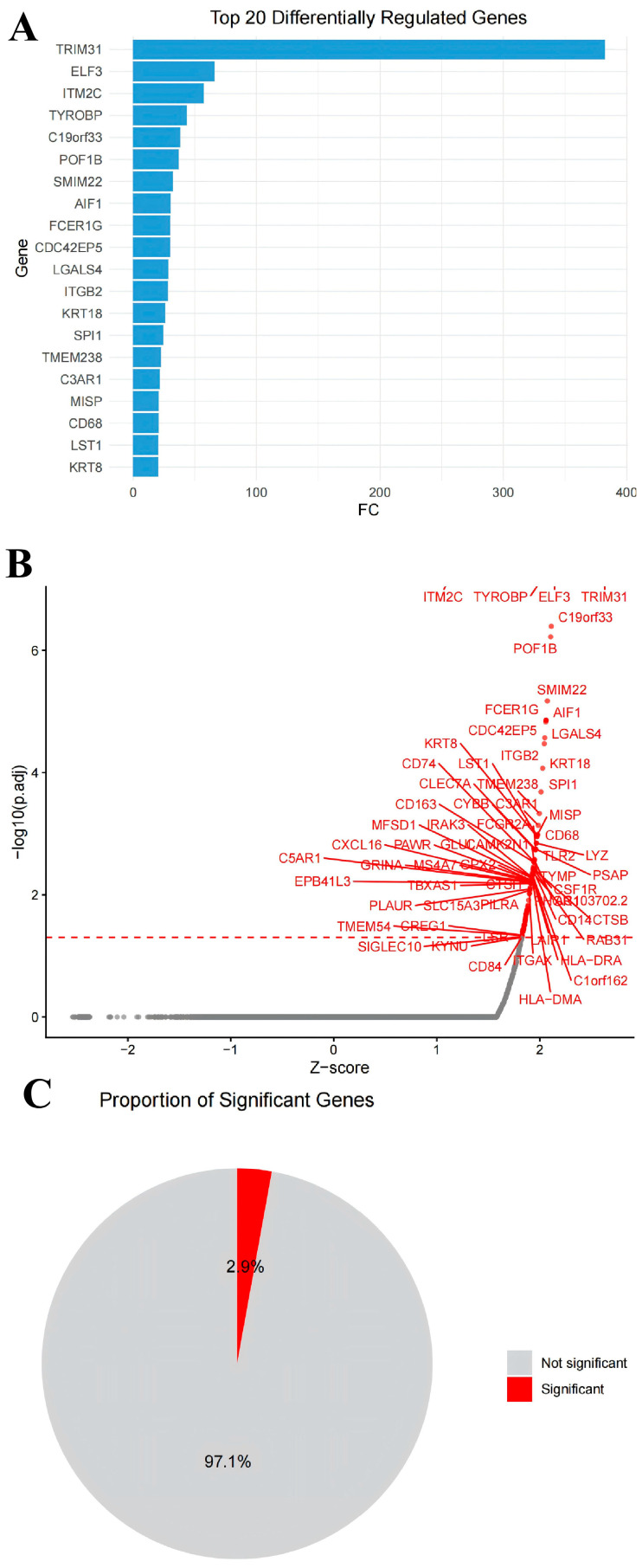
After simulating the knockout of the *LTB4R* gene, the genes that were significantly affected. (**A**) Bar chart of the genes significantly affected by *LTB4R* knockout. (**B**) Volcano plot of the genes significantly affected by *LTB4R* knockout. (**C**) Pie chart of the proportion of genes significantly affected by *LTB4R* knockout.

In order to further explore the molecular mechanism of the *LTB4R* gene in the pathological process of CRC, we conducted a single-cell omics simulation of gene knockout analysis. The results illustrated that after the *LTB4R* gene was knocked out, a total of 106 genes were significantly affected (as listed in Table S4). Among them, the genes most affected include *TRIM31, ELF3, ITM2C, ITGB2, AIF1,* etc. (Figure 7A,B). Furthermore, the genes that were significantly affected accounted for 2.9% of the total number of genes (Figure 7C).

**Figure 8:**
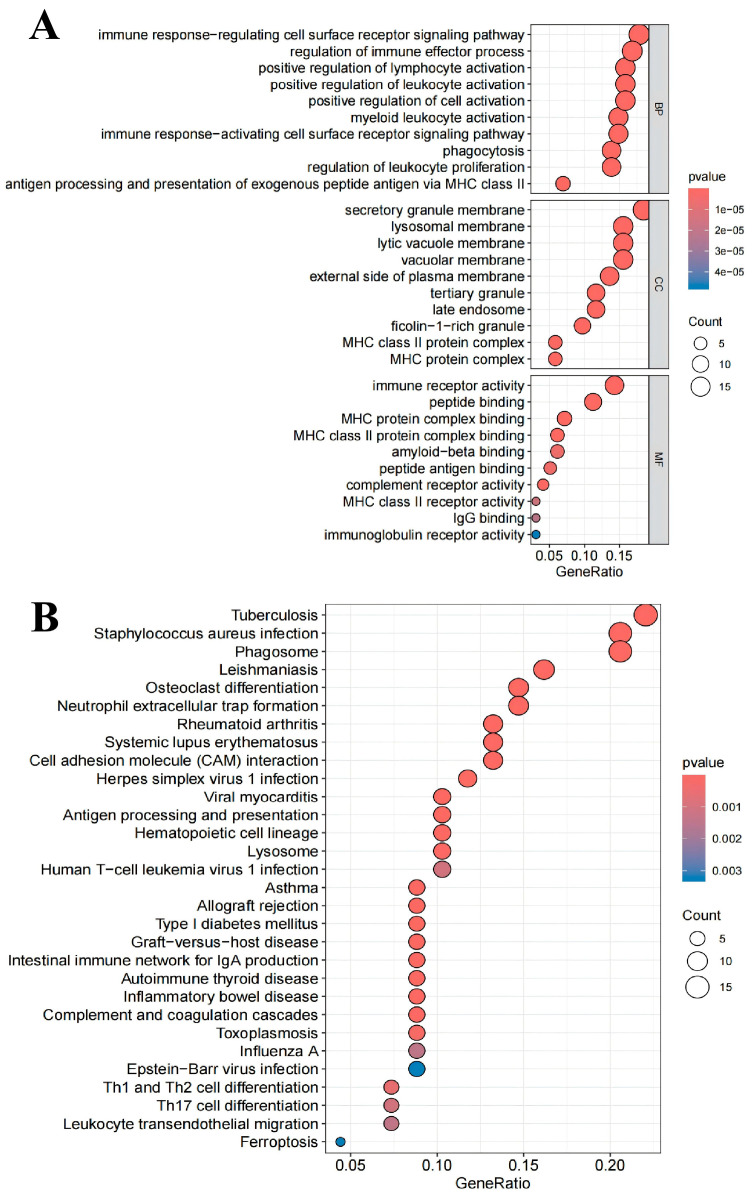
Enrichment analysis of genes significantly affected after *LTB4R* gene knockout simulation. (**A**) GO enrichment analysis diagram; (**B**) KEGG enrichment analysis diagram.

Subsequently, the identified genes were subjected to GO and KEGG enrichment analyses. The GO enrichment analysis suggested that 106 genes were highly correlated with immune response-regulating cell surface receptor signalling pathway, immune receptor activity, regulation of immune effector process, secretory granule membrane and peptide binding (Figure 8A). The pathways enriched by the KEGG analysis include tuberculosis, staphylococcus aureus infection, phagosome, leishmaniasis and osteoclast differentiation (Figure 8B). The above pathways were all related to immune responses, inflammatory responses, and the intestinal microbiota, indicating that knocking out the *LTB4R* gene can affect these tumour-related pathways.

### 2.5. LTB4R Gene Can Serve as a Diagnostic Marker for Colorectal Cancer

**Figure 9:**
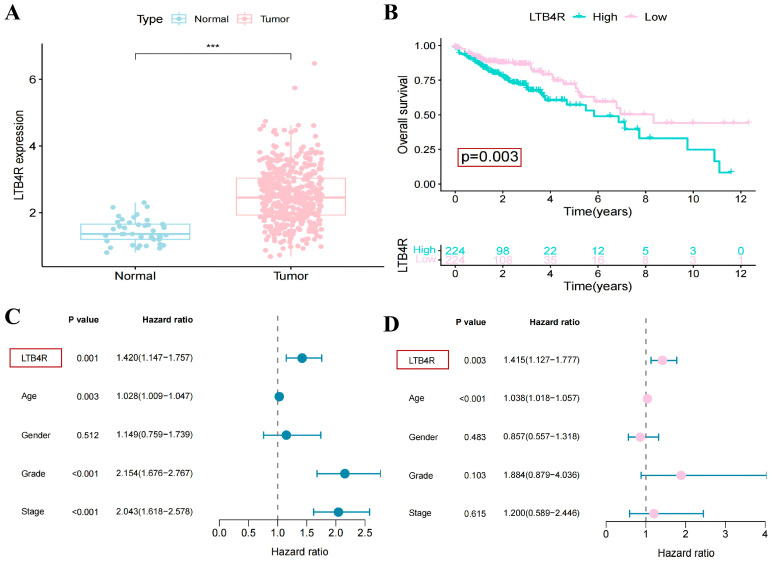
The *LTB4R* gene can serve as a potential biomarker for colorectal cancer. (**A**) The expression level map of *LTB4R* in tumour tissues and normal tissues in single-cell sequencing data. (**B**) Overall survival analysis graph (Kaplan–Meier curve) for the high and low expression groups of the *LTB4R* gene. (**C**) Univariate survival analysis forest plot of *LTB4R* gene expression, age, gender, and grade factors. (**D**) The multivariate survival analysis forest plot of *LTB4R* gene expression, age, gender, and grade factors. *** (*p* < 0.001).

**Figure 10:**
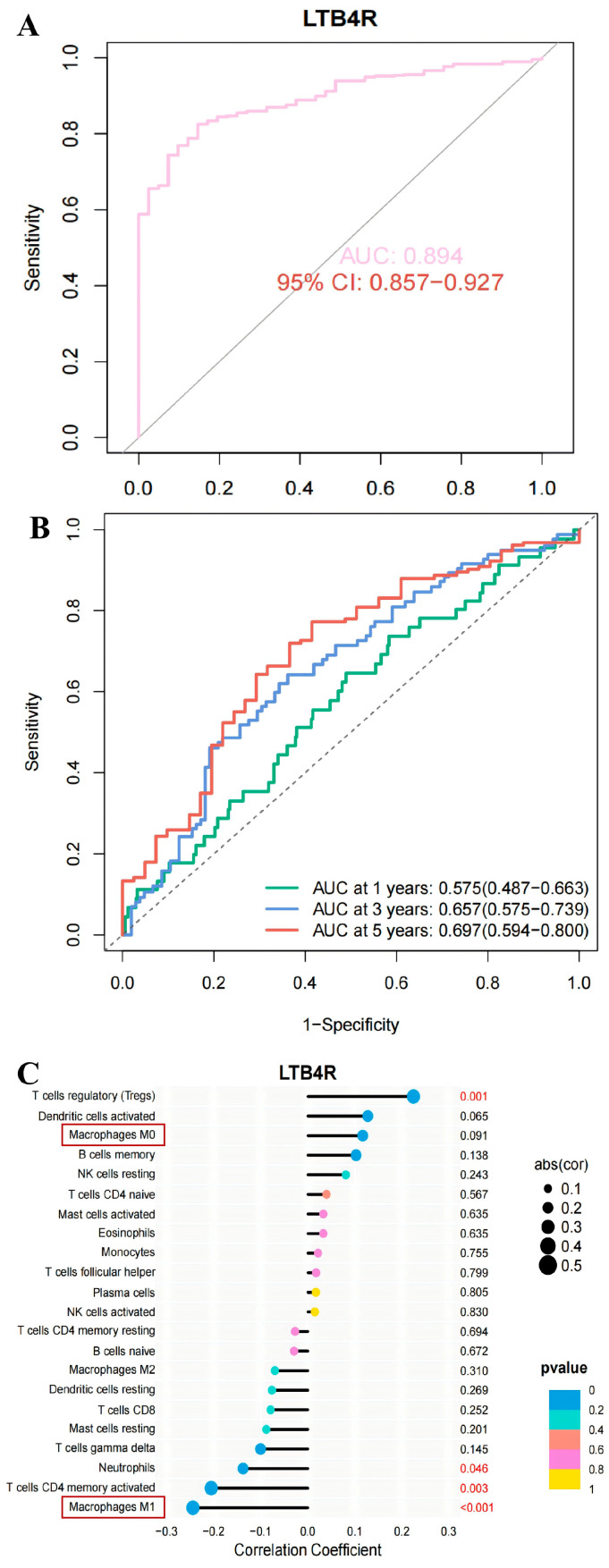
The *LTB4R* gene can serve as a potential biomarker for colorectal cancer. (**A**) The ROC curve for predicting the overall survival rate of CRC patients based on the expression level of *LTB4R*. (**B**) The time-dependent ROC curve for predicting overall survival in CRC patients based on *LTB4R* expression levels. (**C**) The correlation analysis diagram of the *LTB4R* gene and immune cells.

After clarifying the distribution of immune cells carrying the *LTB4R* gene and the influencing pathways, we further evaluated whether this gene has the potential to serve as a diagnostic marker for CRC. The results showed that the *LTB4R* gene was significantly upregulated in CRC tumour tissues, and its high expression was associated with a lower survival rate of the patients (Figure 9A,B). We also drew single-factor survival analysis forest plots and multi-factor survival analysis forest plots to illustrate the impact of factors such as *LTB4R* gene expression, age, gender, and grade on the prognosis of CRC patients. The results showed that both the single-factor survival analysis results (OR = 1.42 (1.147–1.757)) and the multi-factor survival analysis results (OR = 1.415 (1.127–1.777)) indicated that the *LTB4R* gene was a high-risk factor for the prognosis of CRC (Figure 9C,D). Furthermore, the ROC curve of *LTB4R* (with an AUC of 0.894) indicates that it has a good diagnostic potential (Figure 10A,B). Furthermore, the analysis of immune cell correlations revealed that the *LTB4R* gene was significantly negatively correlated with M1-type macrophages (Figure 10C). Based on the analysis results of single-cell sequencing data, it is speculated that *LTB4R* may exert a tumour-promoting effect by regulating M1-type macrophages.

## 3. Discussion

This study employed bioinformatics approaches to analyse multi-omics data for screening diagnostic biomarkers of CRC and elucidated its underlying molecular mechanisms. Specifically, the work first identified the candidate gene *LTB4R* with biomarker potential in peripheral blood by analysing sequencing and clinical data from the TCGA database. Subsequently, the distribution of *LTB4R* in immune cells and its negative correlation with M1 macrophages were revealed through the analysis of single-cell sequencing data and in silico gene knockout modelling. Finally, we confirmed the overexpression of *LTB4R* in CRC by analysing peripheral blood RNA sequencing data from the GEO database, further supporting its potential as a diagnostic biomarker.

The analytical methods adopted in this study were designed to leverage the unique advantages of various omics datasets. First, the sequencing and clinical data from the TCGA database provided a foundation for survival and prognostic analyses. Second, single-cell sequencing data from the GEO database more clearly depicted the expression of *LTB4R* in different immune cell populations. Additionally, in silico gene knockout modelling based on single-cell sequencing data enabled the identification of signalling pathways affected by this gene in CRC without the need for experimental intervention. Furthermore, as the aforementioned data were derived from the sequencing of solid tumour tissues, which are clinically challenging to obtain and unsuitable for routine screening, we also analysed peripheral blood sequencing data from CRC patients and healthy controls. The results demonstrated that *LTB4R* was similarly overexpressed in the peripheral blood of CRC patients, highlighting its potential as a highly clinically accessible diagnostic biomarker.

The *Leukotriene B4 receptor 1 (LTB4R)* gene is located on human chromosome 2 (2q13), and its encoded LTB4R protein is a G protein-coupled receptor (GPCR) belonging to the leukotriene B4 receptor family [13]. It mediates inflammatory responses and immune regulation by specifically binding to *leukotriene B4 (LTB4)* and has gained increasing attention due to its crucial role in malignant tumours [14]. The protein consists of seven transmembrane domains, with the N-terminus located extracellularly for ligand binding and the C-terminus intracellularly involved in G protein signalling pathway activation [13]. It plays a pivotal role in cell migration, inflammatory mediator release, and immune cell chemotaxis. Studies have shown that the aberrant activation of *LTB4R* is closely associated with the development of various inflammation-related diseases and the formation of the tumour microenvironment [15,16,17,18]. Its overexpression can promote tumour-associated macrophage infiltration, angiogenesis, and metastatic niche formation. Additionally, the methylation level of the *LTB4R* gene is significantly correlated with the early occurrence of breast cancer, and its expression profile changes can serve as a potential biomarker for assessing tumour inflammatory microenvironment activity [19,20,21]. Multi-omics analyses in CRC have confirmed that *LTB4R*, along with chemokine genes such as *CCL20* and *CXCL8*, constituted a characteristic molecular signature of inflammation-related molecular subtypes [22]. This molecular classification model can effectively distinguish CRC patients with different prognostic risks, providing new insights for early screening and stratified treatment. Our results demonstrated that *LTB4R* was highly expressed in both CRC tissues and peripheral blood samples from CRC patients. Furthermore, survival and prognostic analyses indicate that the *LTB4R* gene holds potential as a diagnostic biomarker.

M1 macrophages play a pivotal antitumour role in CRC by recognising tumour-associated damage signals (e.g., *IFN-γ* and *LPS*), thereby promoting *iNOS* and *TNF-α* expression and enhancing the inflammatory microenvironment to suppress tumour growth [23]. Moreover, M1 macrophage infiltration is associated with improved prognosis in CRC patients, highlighting their potential as an immunotherapeutic target for CRC [24,25]. Our results demonstrate that *LTB4R* is predominantly expressed in macrophages, T cells, and monocytes. Correlation analysis further confirms a significant negative correlation between this gene and M1 macrophages. Therefore, in conjunction with prior research findings, we hypothesise that *LTB4R* may promote tumour progression by modulating M1 macrophage activity.

However, this study primarily relies on the analysis of omics data, necessitating validation through additional clinical cohorts and experimental studies. Furthermore, the simulation of gene knockout based on single-cell sequencing data also requires more in-depth molecular biology experiments for verification, in order to facilitate the transformation of *LTB4R* towards clinical applications.

## 4. Materials and Methods

### 4.1. Download Genomic and Clinical Data of Colon Adenocarcinoma (COAD)/Rectum Adenocarcinoma (READ) and Screen Key Genes

The genomic and clinical data of COAD and READ were retrieved from the TCGA database (https://portal.gdc.cancer.gov/) [26]. The COAD dataset included 461 samples and the corresponding clinical information. The READ dataset included 172 samples and the corresponding clinical information. First, we merged the data through the “TCGAbiolinks” package. Then, the mRNA expression data and the clinical data of CRC were sorted out. Differential expression analysis (DEGs) was conducted using the “limma” and “DESeq2” software (version 1.44.0) packages. Survival analysis and independent prognostic analysis were performed using the “survival” and “survminer” packages. A heat map of differentially expressed genes was drawn by using the “pheatmap” (version1.0.13) and “ggplot2” (version 4.0.0) software packages. The R (version 4. 5. 2) and R-studio (2026.04.0-daily-218) are used for data analysis in this work.

### 4.2. Integration of Sequencing Data Sets in Peripheral Blood and Analysis of DEGs

RNA-sequencing datasets for CRC (GSE87211, GSE164191) were retrieved from the GEO database (https://www.ncbi.nlm.nih.gov/geo/, accessed on 13 December 2025). The integrated dataset included 232 normal samples and 276 tumour samples. Data normalisation was initially performed using the “limma” and “sva” packages. Subsequently, differential expression analysis was conducted utilising the “limma” and “DESeq2” packages. Visualisation of the results was achieved employing the “pheatmap” and “ggplot2” packages. The parameter settings during the analysis process were: |log2FC| ≥ 1 and |adjPval| < 0.05.

### 4.3. Download of Single-Cell Sequencing Data, Clustering Analysis, Cell Annotation and Cell Communication Analysis Integration of Sequencing Data Sets in Peripheral Blood and Analysis of DEGs

Single-cell RNA-sequencing datasets for colorectal cancer (GSE231559) were retrieved from the GEO database (https://www.ncbi.nlm.nih.gov/geo/, accessed on 13 December 2025) [27]. This scRNA-seq dataset consisted of three healthy intestinal mucosal tissues from normal individuals as the control group, and six tumour tissues from CRC patients as the experimental group. Subsequently, R was used to conduct differential expression gene analysis for the single-cell sequencing data, survival analysis of the DEGs, independent prognostic analysis of the DEGs, and cell communication analysis. The R packages used include “DESeq2”, “monocle”, “celldex”, “SingleR”, “scrapper”, “assertthat”, “svglite”, “circlize”, “ggalluvial”, “igraph”, “patchwork”, “NMF” and “SCpubr”. The parameter settings during the analysis process were |log2FC| ≥ 1 and |adjPval| < 0.05.

### 4.4. Single-Cell Simulation of Gene Knockout Analysis (scTenifoldKnk) and Gene Enrichment Analysis

The scTenifoldKnk package was used to conduct the *LTB4R* gene knockout analysis on the scRNA-seq data [28]. After performing principal component analysis normalisation, 20 independent simulations were conducted, each containing 1500 randomly selected cells. From each simulation, the top 100 genes ranked by |fold change| were extracted, and the genes that appeared ≥5 times in each simulation were defined as stable knockout response genes. Subsequently, we conducted Gene Ontology (GO) enrichment analysis and Kyoto Encyclopedia of Genes and Genomes (KEGG) (https://www.genome.jp/kegg/, accessed on 13 December 2025) enrichment analysis on the genes selected that were related to the knockout of *LTB4R*.

### 4.5. Analyse the Expression Level of the LTB4R Gene in Single-Cell Sequencing Data, as Well as the Survival Curve, Prognostic Analysis, ROC Curve, and Immunocyte Correlation Related to the LTB4R Gene

Using packages such as “limma”, “ggplot2”, and “ggpubr”, the expression levels of *LTB4R* in the tumour group and the normal group were analysed in the single-cell sequencing data. Subsequently, based on the clinical data analysis, the *LTB4R* gene was correlated with the survival curve of CRC. And, using packages such as “timeROC”, “survminer”, “survival” and “reshape2”, univariate prognostic analysis, multivariate prognostic analysis, survival analysis, and correlation analysis between *LTB4R* and immune cells were conducted. The parameter settings were |adjPval| < 0.05. All the codes used in this study were presented in Table S5.

## 5. Conclusions

This study integrated the unique advantages of multiple omics datasets through bioinformatics analysis, suggesting that *LTB4R* may serve as a potential diagnostic marker for CRC. Our results indicated that *LTB4R* was significantly upregulated in both CRC tissues and the peripheral blood samples of CRC patients. By leveraging single-cell sequencing-based gene knockout simulations, we further explored the molecular mechanisms underlying *LTB4R* function, suggesting that it may be involved in tumour progression, possibly through the negative regulation of M1-type macrophage polarisation. To our knowledge, this study provides the first systematic evaluation of *LTB4R* as a potential non-invasive peripheral blood diagnostic marker for CRC, providing a new perspective for early diagnosis and clinical screening strategies.

## Data Availability

Dataset available on request from the authors. The raw data supporting the conclusions of this article will be made available by the authors on request.

## Supplementary Materials

The following supporting information can be downloaded at: https://www.mdpi.com/article/10.3390/ijms27062575/s1.

## Abbreviations

The following abbreviations are used in this manuscript:

CRC	Colorectal cancer
scRNA-seq	Single-cell RNA sequencing
LTB4R	Leukotriene B4 receptor 1
scTenifoldKnk	Simulated gene knockout analysis
TME	Tumour microenvironment
DEGs	Differentially expressed genes
ROC	Receiver operating characteristic
COAD	Colon adenocarcinoma
GO	Gene Ontology
KEGG	Kyoto Encyclopedia of Genes and Genomes
GPCR	G protein-coupled receptor
