# Supplementary material for: Multi-Omics Analysis Identified LTB4R as a Peripheral Blood Diagnostic Biomarker for Colorectal Cancer
Source: Int J Mol Sci. 2026 Mar 11;27(6):2575. doi: 10.3390/ijms27062575 (PMC13026665; doi:10.3390/ijms27062575)
Supplement: Supplementary file 1 [file ijms-27-02575-s001.zip › ijms-4156564-supplementary.pdf]

Supplementary Materials

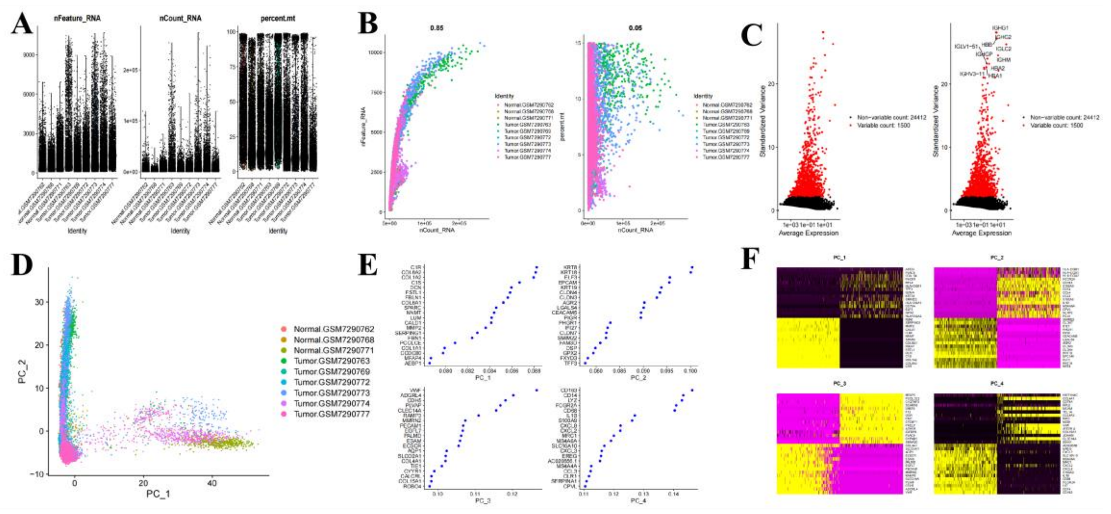

Figure S1. Graphs presenting the results of single-cell sequencing data analysis. (A) Quality control chart for single-cell sequencing data. (B) Graph showing the correlation between sequencing depth and the number of genes (left graph) (0.85 indicates a strong correlation), and the graph on the right shows the correlation between sequencing depth and the number of mitochondrial genes (right graph) (0.05 indicates almost no correlation). (C) Volcano plot of differentially expressed genes in single-cell sequencing analysis. (D) Scatter plot of principal component analysis in single-cell sequencing analysis. (E) Correlation diagrams of the 4 different principal components in single-cell analysis with genes. (F) Heatmap of the 4 principal component analyses in single-cell analysis.

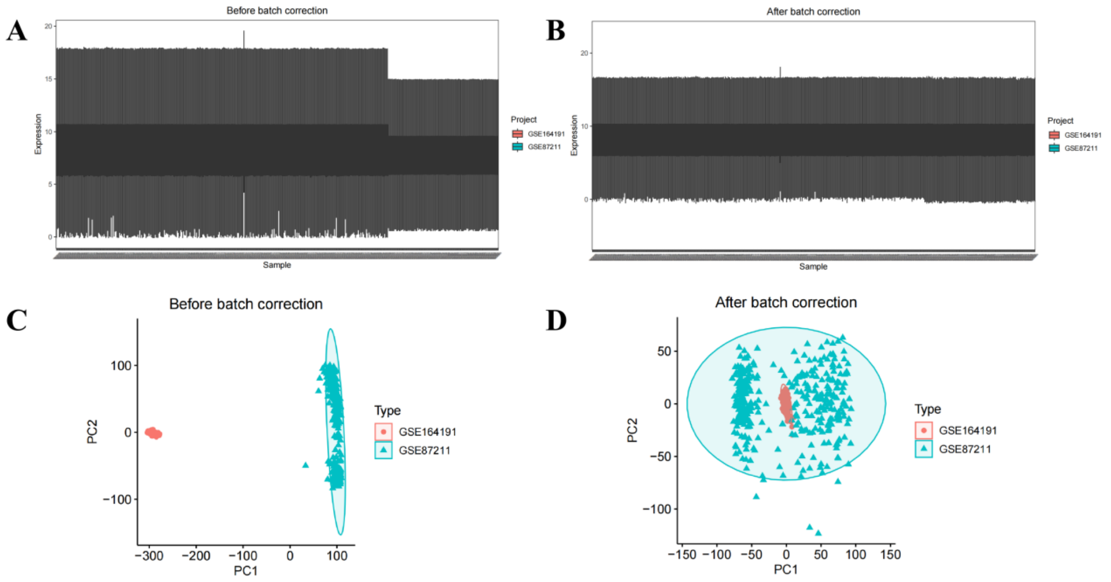

Figure S2. (A) (B) The quality control analysis results before standardization of the two GSE datasets. (C) (D) The quality control analysis results after standardization of the two GSE datasets.

Table S1. DEGs between the CRC group and the Control group;.

Table S2. The DEGs identified from survival analysis;.

Table S3. Independent prognostic DEGs of CRC; Table S4: The DEGs affected by scTenifoldKnk; Table S5: The code used in this work.
